# Human pluripotent stem cells as alternative models to study trophoblast development

**DOI:** 10.3389/fphys.2013.00374

**Published:** 2013-12-17

**Authors:** Youssef Hibaoui, Anis Feki

**Affiliations:** ^1^Stem Cell Research Laboratory, Department of Obstetrics and Gynecology, Geneva University HospitalsGeneva, Switzerland; ^2^Service de gynécologie obstétrique, HFR Fribourg - Hôpital cantonalFribourg, Switzerland

**Keywords:** embryonic stem cells, induced pluripotent stem cells, trophoblast differentiation, implantation, pregnancy, placental disorders

The trophoblast, which is derived from the extraembryonic trophectoderm, is the first cells differentiated from the pre-implantation mammalian embryos. In this regard, two differentiation pathways are critical for the survival and development of the embryo *in utero*. In the first one, cytotrophoblasts (CTBs) fuse into a layer of syncytiotrophoblasts that covers the chorionic villi. These cells are responsible for hormone production, nutrient and gas exchanges between the mother and the developing fetus. In the second one, CTBs in the anchoring chorionic villi invade the uterus and its blood vessels establishing the maternal-fetal interface (Red-Horse et al., 2004; Roberts and Fisher, 2011). Dysfunction of these differentiation pathways is associated with a wide range of human pregnancy complications such as infertility, preterm birth, pre-eclampsia, intrauterine growth restriction and aneuploidies (Red-Horse et al., 2004).

To date, most of the insights on placental trophoblast development have been gained through transgenic mouse models and mouse trophoblast stem cells with placental defects (Kunath et al., 2004). Despite similarities to human phenotype, mouse models have several drawbacks and cannot integrate the specificities of human placentation. To overcome the unfeasibility of performing *in vivo* experimentation in humans, cell lines derived from human choriocarcinoma or virally transformed human trophoblast cell lines are widely used to investigate the mechanisms involved in trophoblast differentiation (Hannan et al., 2010). However, general limitations of using cell lines exist such as their genetic background and the potential changes acquired after their transformation and during their establishment in culture. Alternatively, primary trophoblast cultures have been derived from human placentas at the first trimester or at term of pregnancy. While these cells might be an excellent model to study trophoblast differentiation, they are often difficult to obtain and do not proliferate in culture. In addition, these cells are already committed to the trophoblast lineage and therefore are not amenable to study early trophoblast development.

The discovery that human pluripotent stem cells (hPSCs) can be differentiated into trophoblast cells through bone morphogenetic proteins (BMPs) (Xu et al., 2002) has opened up a new field of investigation in human trophoblast developmental biology and disease (Ezashi et al., 2012; Golos et al., 2013). Thomson's group was the first showing trophoblast differentiation of human embryonic stem cells (hESCs) following BMP4 treatment (Xu et al., 2002). Then, the same group has shown that in the presence of high concentrations of FGF2, BMP4 treatment results in the differentiation of hESCs into mesendoderm rather than trophoblast. They demonstrated in particular that FGF signaling switches BMP4-induced differentiation of hESCs into mesendoderm via the maintenance of *NANOG* expression through MEK/ERK pathway (Yu et al., 2011). Thereafter, other studies have emphasized the need of dual inhibition of FGF signaling (with PD173074 or SU5402) and TGF-β/ACTIVIN/NODAL signaling (with SB431542 or A83-01) to support trophoblast induction specifically (Sudheer et al., 2012; Amita et al., 2013). Under these conditions, BMP4-induced differentiation leads to the emergence of both chorionic gonadotrophin β-secreting syncytiotrophoblasts with a villous CTB phenotype and HLA-G^+^ trophoblast cells with invasive properties as the extravillous CTBs (Marchand et al., 2011; Sudheer et al., 2012; Amita et al., 2013; Li et al., 2013). Furthermore, by their ability to recapitulate the early and late steps of trophoblast development, hESCs should become valuable tools to study the cellular and molecular mechanisms involved in trophoblast cell maintenance, specification and differentiation.

Since 2007, the generation of human induced pluripotent stem cells (hiPSCs) has allowed researchers to study the development and/or progression of several pathologies including neurological diseases [reviewed in Hibaoui and Feki (2012)]. It is undoubted that similar approaches using patient-specific iPSCs will offer the opportunity to reproduce normal and pathological trophoblast development. Moreover, hiPSCs exhibited additional advantages compared to hESCs. First, hiPSCs do not require the use of human embryos, which makes their use in basic research less ethically controversial. They are also technically easier to obtain. Last but not least, they offer the opportunity to generate iPSC lines specific for patients with pregnancy complications. Of note, the recent discovery that iPSCs generated by *in vivo* reprogramming of adult mouse cells efficiently differentiate into the three germ layers and extraembryonic tissue (Abad et al., 2013), may improve our understanding of extraembryonic lineage development and placenta-associated disorders.

In conclusion, while further studies are needed to improve the efficiency of hPSC differentiation into trophoblast cells, we believe that this technology provides a promising alternative model to study trophoblast development. In fact, understanding and elucidating the mechanisms regulating human trophoblast development using hPSCs is critical not only in basic research but also in clinical medicine. It could provide novel insights into blastocyst formation, implantation and placentation processes in humans. Thus, patient-specific hPSCs will also open up new exciting avenues for the identification of genetic and biochemical markers that could be useful for the prediction and management of pregnancy complications such as pre-eclampsia, intrauterine growth restriction and aneuploidies. The coming years will tell whether these cells fulfil their promise.
